# Brain Activations Related to Saccadic Response Conflict are not Sensitive to Time on Task

**DOI:** 10.3389/fnhum.2015.00664

**Published:** 2015-12-02

**Authors:** Ewa Beldzik, Aleksandra Domagalik, Halszka Oginska, Tadeusz Marek, Magdalena Fafrowicz

**Affiliations:** ^1^Department of Cognitive Neuroscience and Neuroergonomics, Institute of Applied Psychology, Jagiellonian University in KrakowKrakow, Poland; ^2^Neurobiology Department, Malopolska Centre of Biotechnology, Jagiellonian University in KrakowKrakow, Poland

**Keywords:** fMRI, saccades, response conflict, time on task, pre-SMA

## Abstract

Establishing a role of the dorsal medial frontal cortex in the performance monitoring and cognitive control has been a challenge to neuroscientists for the past decade. In light of recent findings, the conflict monitoring hypothesis has been elaborated to an action-outcome predictor theory. One of the findings that led to this re-evaluation was the fMRI study in which conflict-related brain activity was investigated in terms of the so-called time on task effect, i.e., a linear increase of the BOLD signal with longer response times. The aim of this study was to investigate brain regions involved in the processing of saccadic response conflict and to account for the time on task effect. A modified spatial cueing task was implemented in the event-related fMRI study with oculomotor responses. The results revealed several brain regions which show higher activity for incongruent trials in comparison to the congruent ones, including pre-supplementary motor area together with the frontal and parietal regions. Further analysis accounting for the effect of response time provided evidence that these brain activations were not sensitive to time on task but reflected purely the congruency effect.

## Introduction

Response conflict occurs when a task simultaneously activates more than one response tendency. According to the “conflict monitoring hypothesis” (Botvinick et al., 2001), the region engaged in monitoring the occurrence of such competition in action selection is the dorsal medial frontal cortex (dMFC), something that has been widely confirmed (see reviews by Botvinick et al., 2004; Botvinick, 2007). However, recent studies have provided evidence that dMFC activity can be associated with the “time on task effect”, i.e., a linear increase of the BOLD signal with longer response times (RTs), instead of the conflict processing itself (Carp et al., 2010; Grinband et al., 2011b; Weissman and Carp, 2013). These findings raised some doubts regarding the general concept of conflict hypothesis and called for a profound examination of the conflict-related activity evoked by other tasks. Thus, in this study, we aimed to examine whether the brain regions associated with saccadic response conflict were also sensitive to time on task.

The last decade of cognitive neuroscience research has consistently indicated that the dMFC, particularly the anterior cingulate cortex (ACC) is the key structure in conflict monitoring (van Veen and Carter, 2002; Botvinick et al., 2004; Botvinick, 2007). Most of these studies used experimental procedures with manual responses, such as Stroop and Eriksen tasks, and compared brain activation associated with high-interference stimuli (incongruent trials) and low-interference ones (congruent trials). Greater ACC activation for incongruent trials in comparison with congruent trials was linked to reactive adjustments in cognitive control (Botvinick, 2007). According to the authors, this mechanism provides the neural basis for an empirically observed bias toward tasks and strategies that involve efficient information processing.

However, a multi-study fMRI analysis by Yarkoni et al. (2009) showed that activation in lateral and medial frontal regions increased linearly as a function of RT, that is, with the time on task. Later, the study by Grinband et al. (2011b) presented serious challenges to the conflict monitoring hypothesis. The authors showed that response duration affects the dMFC activation to such an extent that when fast incongruent and slow congruent trials were selected, the activation of dMFC inverted, being stronger for congruent trials in comparison with incongruent ones. Another study by Carp et al. (2010) implemented a novel approach in post-processing fMRI data analysis and showed that removing the effect of RT eliminates conflict-related activity in dMFC. Thus, this “inconvenient” relationship between RT and BOLD signal (Domagalik et al., 2014) calls for a re-examination of the fMRI data analyses by those who have implemented other conflict tasks or will implement them in the future.

Cognitive tasks with oculomotor responses comprise trials with various cases of stop-the-saccade cues (Curtis et al., 2005), invalid-direction cues (Nakamura et al., 2005) or change-of-plan cues (Nachev et al., 2008). Thus, saccadic response conflict is evoked by altering, rather than distracting, the planned action with an irrelevant feature. Both neurophysiological (Ito et al., 2003; Isoda and Hikosaka, 2007) and fMRI studies (Nachev et al., 2005) using saccadic tasks indicate that the brain region responsible for successful switch to a controlled alternative action is the pre-supplementary motor area (pre-SMA). Rushworth et al. (2004) suggested a functional distinction between pre-SMA and ACC—the two adherent brain regions within dMFC. According to the authors, pre-SMA is involved in the selection of action sets, whereas ACC has a role in relating actions to their consequences, both positive reinforcement outcomes and errors. The latter conclusion was supported by the recent computational (Alexander and Brown, 2011), EEG (Beldzik et al., 2015) and fMRI studies (Jessup et al., 2010; Jahn et al., 2014).

In this study, we aimed at verifying whether conflict-related activity in pre-SMA is sensitive to the time-on-task. Similarly to the study by Nakamura et al. (2005), we used a task comprising leftward and rightward, congruent and incongruent cues to evoke the saccadic response conflict. The task was implemented in the event-related fMRI study in order to identify brain areas involved in the conflict processing. We then verified whether these brain activations have changed after controlling for RT variations. Two approaches were chosen to obtain this goal: RT-regression analysis (following Carp et al., 2010) and the comparison of slow and fast responses after event-triggered averaging (following Grinband et al., 2011b). Due to the fundamental role of cue in programming the saccade direction (Dassonville et al., 1995), it is expected that the inhibition and spatial re-orientation, which lead to conflict, are present on every incongruent trial regardless of RT variations. Thus, we hypothesized that conflict-related activity is not prone to the time on task effect i.e., it does not vary with RT.

## Materials and Methods

### Participants

Participants were 23 women (mean age 23.4 ± 2.0 years) all of whom met the magnetic resonance inclusion criteria and experiment requirements: right-handed, right-eyed dominant, normal or corrected-to-normal vision, no physical and psychiatric disorders. They were all non-smokers and drug-free. Participants were trained to ensure familiarity with MR scanner and with the experimental task. This study was carried out in accordance with the recommendations of Bioethics Commission at the Jagiellonian University. All subjects gave written informed consent in accordance with the Declaration of Helsinki.

### Experimental Task and Procedures

**Figure 1:**
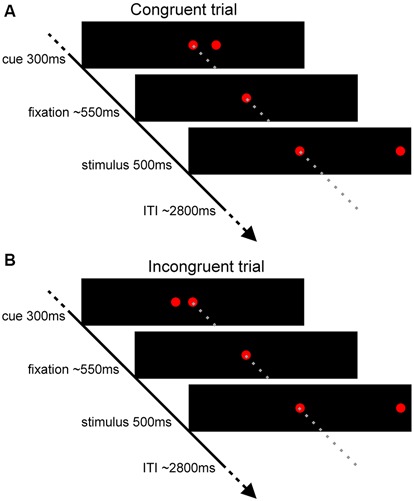
**Experimental task used in the study.** Scheme presenting **(A)** the congruent trials and **(B)** the incongruent trials.

The experimental task was the spatial cueing paradigm (Posner, 1980). Participants were instructed to direct their attention and gaze to a target, i.e., stimuli presented on the left or the right side of the fixation point (15 degrees of visual angle), only if they were preceded by a cue (5 degrees of visual angle). The task comprised trials with cues congruent to a target (58%; Figure 1A), incongruent to a target (15%; Figure 1B), and trials without a cue (27%). The proportion of the trials was determined in order to maintain high interference effect (Tzelgov et al., 1992) and optimizing statistical power in fMRI design while retaining task unpredictability (Wager and Nichols, 2003). The trial sequence was pseudo-randomized to counterbalance the presentation of each trial type. Targets were presented for 500 ms, whereas cues for 300 ms. Average interstimulus interval was 550 ms (varying between 300–800 ms in steps of 100 ms), while the average intertrial interval was 2800 ms (varying between 1300 and 4300 ms in steps of 500 ms). To improve sampling rate of the hemodynamic response, the phase of the target was varied relative to the image acquisition (Toni et al., 1991; Josephs et al., 1997) resulting in the final temporal resolution of 100 ms. High temporal resolution was crucial for further event-triggered analysis in which the BOLD signal is interpolated and averaged time-locked precisely to the onset of the events. The trial presentation rate (average trial duration—4150 ms) was considered to be sufficiently low given this temporal resolution as well as the linearly additive nature of hemodynamic response functions at the short intertrial intervals (Dale and Buckner, 1997; Burock et al., 1998; Soon et al., 2003). Participants performed the task during four magnetic resonance scanning sessions conducted in one day. Each session comprised 598 trials and lasted about 40 min.

### Eye Tracking

The position of an eye was monitored using a Saccadometer Research MRI system (Ober-Consulting, Poland). The system registers right eye movements using direct near-infrared technology. It has 500 Hz sampling frequency, measuring range ±20 degrees of visual angle and average spatial resolution of 15′ (this accuracy was verified in the scanner before the experiment). Stimuli generated by red laser diodes were presented in the horizontal axis on a panel integrated with saccadometer system attached to the subject’s head approximately 3 cm from the subject’s eyes. There were five diodes on the panel: central diode for fixation, left and right diodes for cue stimuli and targets. A calibration procedure before each session was conducted—the participants looked three times at each stimulus for a 1 s period. Eye-tracking data were analyzed using Research Analyzer software (Ober-Consulting, Poland). Saccades were detected with the velocity criteria: the beginning of a saccade was marked when an eye movement was faster than 5 degrees/s (Edelman et al., 2006) and this movement was classified as a saccade only when its velocity reached 90 degrees/s (Ethier et al., 2008). SRT was defined as a difference between target appearance and the beginning of saccade. Reaction was classified as correct when a saccade was programmed in the correct direction and reached a target during its presentation.

### fMRI Data Acquisition

Magnetic resonance imaging (MRI) was performed using a 1, 5T Signa HDxt General Electric (GE Medical Systems, Milwaukee, WI, USA). High-resolution, whole-brain anatomical images were acquired using T1-weighted sequence. A total of 60 axial slices were obtained (voxel dimension = 0.4 × 0.4 × 3 mm^3^; matrix size = 512 × 512, TR = 25.0 ms, TE = 6.0 ms, FOV = 22 × 22 cm^2^, flip angle = 45°) for coregistration with the fMRI data. Functional T2-weighted images were acquired using an echo planar pulse sequence with a TR of 3 s, TE of 60 ms, matrix size of 128 × 128, FOV of 22 × 22 cm^2^, spatial resolution of 1.9 × 1.9 × 6 mm^3^, and flip angle of 90°. Whole brain image was covered with 20 axial slices, taken at an interleaved fashion. Due to magnetic saturation effects, the first three images of each session were excluded from functional analysis.

### Data Analysis

Standard preprocessing procedures were applied to fMRI data with Analysis of Functional NeuroImage software (Cox, 1996). Firstly, each 3D image was time-shifted so that the slices were aligned temporally. After head motion correction with six degrees of freedom, the functional EPI data sets were zero-padded to match the spatial extent of the anatomic scans, and then coregistered using six degrees of freedom. Anatomical and functional images were transformed into a coordinate system of Talairach space (Talairach and Tournoux, 1988) using 12 degrees of freedom. The functional data were then smoothed using a full-width at half-maximum isotropic Gaussian kernel of 8 mm. During scaling procedure, voxels with low-signal intensity located outside the brain were excluded by a clipping function.

The General Linear Model (GLM) was applied for each subject to the concatenated datasets of all sessions (a single beta parameter was derived from the time-course comprising all experimental sessions). Two approaches were used differing between the inclusions of trial-by-trial RT variability in the models. The first, i.e., standard, GLM approach included the following regressors: congruent stimuli followed by the correct responses, incongruent stimuli followed by correct responses, stimuli without a cue, stimuli followed by the erroneous response, six movement parameters and a higher order polynomial accounting for slow drifts in the fMRI time series. Stimuli regressors consisting of impulses were convolved with a double gamma function modelling a prototypical hemodynamic response (Glover, 1999). Next, beta coefficient maps of the regressor of interest, i.e., congruent and incongruent stimuli followed by the correct responses, were created for each individual subject and session. Two contrasts were performed at this level: an additive map (Incong + Cong) and differential map (Incong − Cong). Subsequently, the contrast maps were included in the mixed-effects group statistical analysis. The obtained statistical maps were corrected for multiple comparisons using false discovery rates (FDR).

The second GLM approach was implemented following Carp et al. (2010) in order to remove the effect of RT. This RT-regression analysis enables to correct RT differences between congruent and incongruent trials. In detail, two additional impulse regressors were included in the model: RT for congruent and incongruent trials. RTs were mean-subtracted in units of seconds and convolved with the same double gamma function. As a result, RT-BOLD parameter estimates were yielded for each voxel. The difference between mean RT for incongruent and congruent trials was calculated for each run of each subject. Then, RT parameter estimates of congruent trials were multiplied by this quantity. This product was added to parameter estimates for congruent trials creating a RT-equated congruent parameter estimates. Comparison of the activity for incongruent trials to activity for RT-equated congruent trials resulted in conflict equated (ConflictEQ) map obtained for each subject and each session. Subsequently, the ConflictEQ maps were included in the mixed-effects group statistical analysis. The statistical map was corrected for multiple comparisons using FDR.

Next, an event-triggered averaging approach was performed in order to account for the time on task effect (Grinband et al., 2011b). The analysis was applied to the fMRI data derived from every cluster in the conflict map obtained with the standard GLM model. In details, fMRI time series were extracted for each subject and session from these regions of interest. Only correct trials with RTs greater than 80 ms were included in the analysis in order to eliminate anticipatory saccades (Fischer et al., 1997). Similarly to Grinband et al. (2011b), three separate comparisons were conducted: (1) all congruent and all incongruent trials; (2) equalized trials, i.e., congruent and incongruent trials with RT ± 25 ms of each subject’s global median; and (3) slow congruent trials (RT greater that the global median) and fast incongruent trials (RT smaller that the global median). The extracted time series were interpolated to 50 ms resolution, segmented into stimuli-locked time epochs and averaged separately for each RT comparison. The mean response was then averaged across subjects and paired *t*-test was performed to test for the differences in the size of the BOLD response between congruent and incongruent trials.

## Results

Behavioral results for the incongruent and congruent trials are presented in Table 1. The results obtained indicate that the conflict was induced by the task: an average RT for correct incongruent trials was significantly longer than for correct congruent trials (*t*_(1,22)_ = 5.42; *p* < 0.001), whereas the error rate was greater after the incongruent stimuli in comparison to congruent (*t*_(1,22)_ = 3.56; *p* = 0.002).

**Table 1:** **Behavioral performance of the task (mean and standard error)**.

Trial type	Accuracy (% incorrect)	Reaction time (ms)
Congruent	2.53 ± 0.62	164.24 ± 3.74
Incongruent	3.88 ± 0.66	184.60 ± 5.34

**Figure 2:**
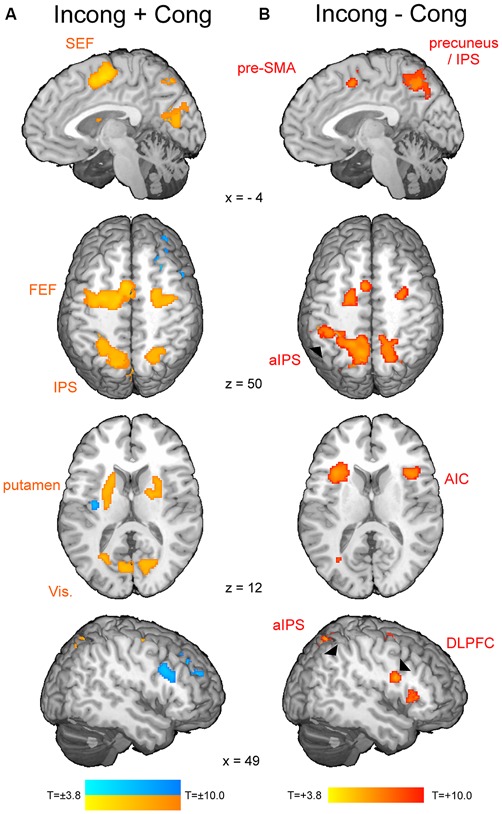
**The fMRI results from the modified spatial cueing paradigm.** Both maps: *T* = 3.799, *p*_cor_ < 0.05. **(A)** Brain regions involved in volitional saccade generation, irrespectively of the congruency effect. SEF, supplementary eye fields; FEF, frontal eye fields; IPS, intraparietal sulcus. **(B)** Brain regions involved in the processing of the saccadic response conflict. pre-SMA, pre-supplementary motor area; aIPS, anterior IPS; AIC, anterior insular cortex; DLPFC, dorsolateral prefrontal cortex.

The *t*-map corresponding to the volitional saccade generation (*t*_(1,22)_ = 3.8, *p*_cor_ < 0.05, cluster size > 50; Table 2; Figure 2A) showed following brain activations: the dMFC with a peak in supplementary eye field (SEF), bilateral frontal eye fields (FEF), intraparietal sulci (IPS) and putamen as well as medial visual cortex. These brain regions can be associated with the stimuli perception, execution of volitional saccades into the chosen direction and they were commonly described in fMRI literature of saccadic tasks (see the review by McDowell et al., 2008). Less common to previous findings, small clusters of deactivation in right superior frontal gyrus, right dorsolateral prefrontal cortex (DLPFC) and left posterior insular cortex were observed.

**Table 2:** **Talairach coordinates (center-of-mass) of the activations related to both trial types (Incong + Cong) obtained from the standard GLM analysis**.

Region	Side	*x*	*y*	*z*	*T*
SEF	M	−3.0	−5.9	54.8	8.08
FEF	R	29.3	−7.8	48.4	4.15
	L	−27.5	−8.4	49.8	6.37
IPS	R	21.3	−65.3	51.1	3.94
	L	−20.8	−61.6	47.2	4.29
Visual cortex	M	2.7	−74.2	14.8	3.92
Putamen	R	23.3	−0.1	15.3	5.08
	L	−21.4	0.6	14.0	4.51
Superior frontal gyrus	R	29.4	33.8	34.0	−3.81
DLPFC	R	45.5	16.4	26.1	−5.29
Posterior insular cortex	L	−36.7	16.8	11.3	−4.80

*Note: Side refers to the location of the activation, where M, medial; L, left and R, right hemisphere. T values refer to the center-of-mass (p*_cor_ *< 0.05). SEF, supplementary eye field; FEF, frontal eye field; IPS, intraparietal sulcus; DLPFC, dorsolateral prefrontal cortex*.

The *t*-map corresponding to the conflict (*t*_(1,22)_ = 3.8, *p*_cor_ < 0.05, cluster size > 30; Table 3; Figure 2B) revealed several brain areas, which showed stronger activation for incongruent than congruent trials. The regions, overlapping with those of volitional saccades map, were the dMFC with a peak in pre-SMA, bilateral medial parts of FEF and IPS extending to precuneus. Few additional brain regions showed stronger activation for incongruent than congruent trials, although they were not found in volitional saccades map. These regions comprise bilateral anterior insular cortices (AIC), bilateral anterior IPS and right DLPFC.

**Table 3:** **Talairach coordinates (center-of-mass) of the activations within the conflict map (Incong vs. Cong) obtained from the two GLM approaches**.

		Conflict map (standard model)				Conflict EQ Carp et al. (2010)			
		*p*_uncor_ < 0.001; *p*_cor_ < 0.05				*p*_uncor_ < 0.001; *p*_cor_ = 0.12			
Region	Side	*x*	*y*	*z*	*T*	*x*	*y*	*z*	*T*
pre-SMA	M	−6.5	3.8	48.5	5.12	−5.1	3.2	48.2	4.79
FEF	R	24.4	−2.3	55.4	4.30	26.2	−5.2	45.0	4.08
	L	−21.6	−7.7	50.0	4.56	−22.1	−8.6	48.1	4.60
IPS	R	17.6	−62.8	53.0	4.41	19.0	−58.8	59.5	4.19
	L	−23.7	−55.4	47.9	4.51	−14.5	−60.5	51.2	5.03
Anterior IPS	R	49.9	41.0	38.0	4.24
	L	−54.7	−39.1	40.2	5.57
AIC	R	40.8	17.0	7.1	4.98
	L	−33.7	16.7	10.6	6.45
DLPFC	R	45.2	4.3	21.2	5.66
Middle occipital	L	−33.4	−73.3	16.6	4.84

*Note: Side refers to the location of the activation, where M, medial; L, left; R, right hemisphere. T values refer to the center-of-mass. pre-SMA, pre-supplementary motor area; FEF, frontal eye field; IPS, intraparietal sulcus; AIC, anterior insular cortex; DLPFC, dorsolateral prefrontal cortex*.

**Figure 3:**
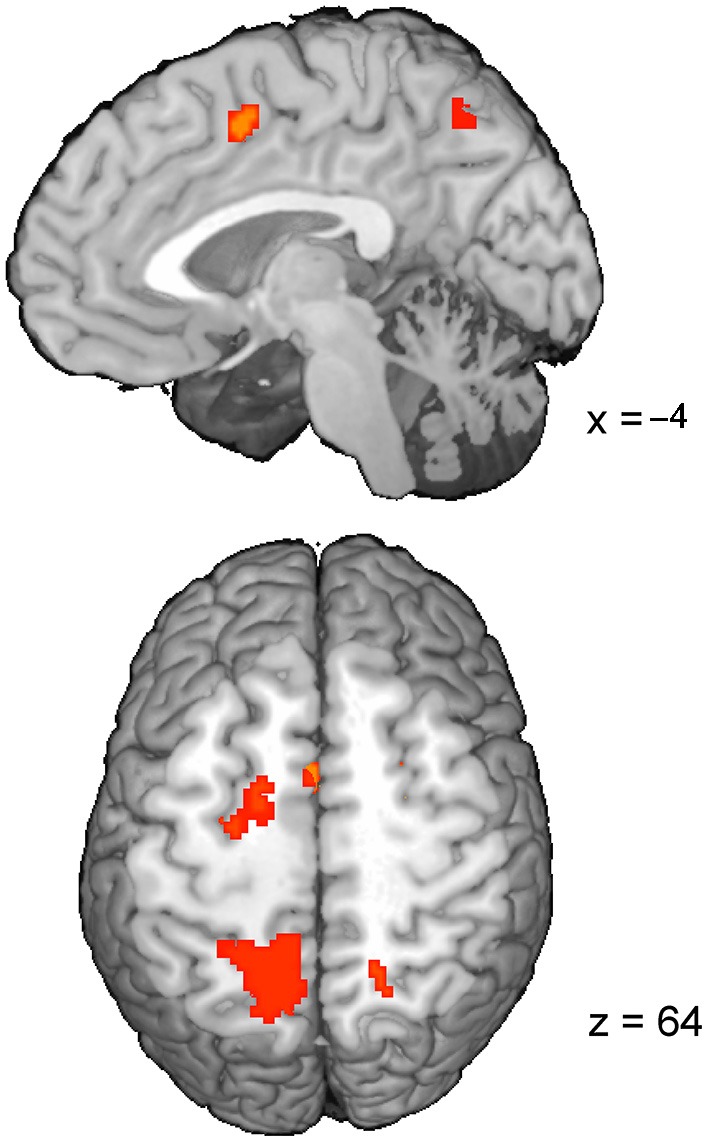
**The ConflictEQ map following Carp et al. (2010).** The approach was introduced in order to account for the effect of RT. A few brain regions, including pre-supplementary motor area, remained under the threshold of *p*_uncor_ < 0.001.

The RT-regression analysis (Carp et al., 2010), resulted in the insignificant (*p*_cor_ = 0.12) ConflictEQ map. However, this map revealed some of the regions listed above, i.e., pre-SMA, bilateral IPS and FEF, to be activated at the threshold *t*_(1,22)_ > 3.8, which corresponds to *p*_uncor_ < 0.001, cluster size > 30 (Table 3; Figure 3).

**Figure 4:**
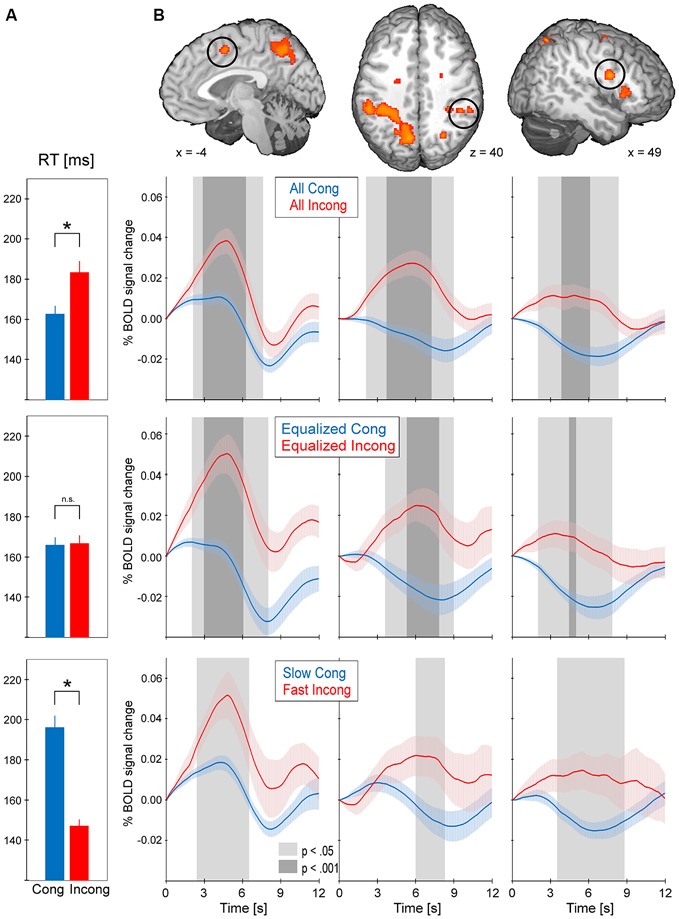
**The results from event-triggered averaging.** BOLD data was extracted from every cluster in the conflict map. BOLD responses were then averaged across the stimuli-locked epochs of 12 s duration and across subjects (shading represents standard error). Trials were selected in three manners: all trials (top), equalized RT (middle) and slow congruent vs. fast incongruent (bottom). **(A)** Average RT for the selected trials; asterisk indicates *p* < 0.001 between the two types of trials. **(B)** BOLD responses for the selected trials in pre-supplementary motor area (pre-SMA), dorsolateral prefrontal cortex (DLPFC) and anterior intraparietal sulcus (aIPS). The remaining clusters of the conflict map showed the same pattern of consistently greater activity for incongruent vs. congruent trials.

Every cluster from the conflict map obtained with the standard GLM approach underwent event-triggered averaging procedures (Figure 4). As expected, comparison between all incongruent and all congruent trials (164.3 ms, standard error = 3.8 ms; RT incong = 184.6 ms, standard error = 5.4; paired *t*-test *p* < 0.001) revealed consistently greater activity for the former type of trials (*p* < 0.001 at the peak of the hemodynamic response in every cluster). Equalized trials were selected ± 25 ms around each subject’s global RT median (average across subjects global median of RT was 168.72 ms, standard error 3.81 ms) and showed no difference in mean RT for the incongruent vs. congruent (RT cong = 165.9 ms, standard error = 4.0 ms; RT incong = 166.8 ms, standard error = 4.0 ms; *p* = 0.19). Event-related BOLD signal for this selection, as before, showed consistently greater activity for incongruent trials in comparison to congruent (at least *p* < 0.05 at the peak of the hemodynamic response in every cluster). The last comparison was obtained for significantly differing in mean RT slow congruent and fast incongruent trials (RT cong = 196.0 ms, standard error = 6.1 ms; RT incong = 147.2 ms, standard error = 3.2 ms; *p* < 0.001). Again, the results obtained showed greater activity for incongruent trials in comparison to congruent (at least *p* < 0.05 at the peak of the hemodynamic response in every cluster except for left AIC).

## Discussion

As soon as the cue appears, observers start programming the saccade and in parallel, start attending to the location of perceptual target (Castet et al., 2006). As a result, the saccade is generated very rapidly after target appearance (here average latency 165 ms). The neural mechanism of this process comprises activations of the visual cortex, IPS, FEF, SEF and putamen (Figure 2A). These brain regions are part of the eye field network, which function involves the perception of stimulus location, initiation and successful generation of saccade into the chosen direction (Domagalik et al., 2012). Incongruent trials induce the saccadic response conflict because the already programmed saccade needs to be reprogrammed. It requires two additional processes: the inhibition of the programmed saccade and the vector inversion (Munoz and Everling, 2004; Domagalik et al., 2012), which is necessary to divert the attention and gaze to the opposite location. In this study, conflict was verified at the behavioral level, i.e., incongruent trials were characterized by significantly longer RTs and higher error rates in comparison with congruent trials. At the neuronal level, a set of distinct brain regions was observed for contrast incongruent vs. congruent trials (Figure 2B).

As expected, the peak of conflict-related activity within dMFC was found in pre-SMA. This finding is in line with the previous studies implementing oculomotor tasks for conflict processing (Nachev et al., 2005; Nakamura et al., 2005; Isoda and Hikosaka, 2007). Pre-SMA enabled switching by first suppressing an automatic unwanted action and then boosting a controlled desired action, thus it is responsible for resolving the response conflict (Isoda and Hikosaka, 2007). Increased activity for incongruent trials in comparison with congruent ones was also observed in bilateral FEF, IPS, AIC, right DLPFC and the precuneus. A study of patients with acute unilateral ischemic lesions of the prefrontal cortex indicated that inhibition of reflexive saccades depends on a circumscribed subregion of the human DLPFC (Ploner et al., 2005). AIC together with DLPFC and dMFC are part of the executive control network, which is responsible for motor inhibition (Berkman et al., 2012). Indeed, this network was linked to withholding a reflexive saccade during the anti-saccade task (Domagalik et al., 2012). Anterior part of IPS and precuneus have been associated with the vector inversion process (Beldzik et al., 2013). Extra involvement of these regions during saccadic conflict resolution can be associated with spatial re-orientation to the target’s location.

Grinband et al. (2011b) found dMFC activity being sensitive to time on task and not response conflict. According to the authors, those findings speak against the conflict monitoring hypothesis (Botvinick et al., 2001). This claim raised debate among cognitive neuroscientists (Brown, 2011; Nachev, 2011; Yeung et al., 2011). Yeung et al. (2011) argued that conflict is measurable by RT variations regardless of the task design; that is, a subject experiences high inference on a congruent trial with slow RT. Thus, according to the authors, dMFC sensitivity to time on task strongly supports the conflict monitoring hypothesis. In response to these comments, Grinband et al. (2011a) pointed out that defining the conflict as “any sensorimotor or cognitive process that lengthens RT” trivializes the idea of conflict. It has been shown that dMFC activity increased linearly with longer RTs regardless of the experimental task or stimulus type (Yarkoni et al., 2009; Carp et al., 2010; Domagalik et al., 2014). Our previous analysis indicated a quadratic increase in dMFC activity with longer RTs even in case of fast and homogenous saccadic reaction to congruent stimuli (Domagalik et al., 2014). The debate was compromised by reestablishing the function of dMFC in performance monitoring and cognitive control. According to the new model by Alexander and Brown (2014), dMFC acts as a predictor of a negative or unexpected response outcome. From the perspective of this model, longer responses can be associated with unexpectedly delayed outcomes, which are marked by a rise in dMFC activity with longer reactions.

In reference to the abovementioned debate, the aim of our study was to verify whether the saccadic conflict-related brain activations are sensitive to time on task. We used two approaches to achieve this goal. First, RT-regression analysis was conducted (Carp et al., 2010) and a ConflictEQ map created (Figure 3). The map did not reach the established threshold of *p* FDR-corrected < 0.05. However, these results do not prove that there was no conflict-related activity. They merely mean that we were unable to find it with this approach. Moreover, five clusters, including pre-SMA, remained under the threshold of *p* uncorrected < 0.001 what suggested that the conflict-related activity could be independent of RT variations and another, valid but less restricted, method could prove it. Hence, event-triggered averaging was applied to a raw signal extracted from brain regions obtained with the standard model without RT (Grinband et al., 2011b) and this approach appeared to be conclusive. Most of the brain regions, significant in the conflict map, showed higher activation for the fast incongruent trials than the slow congruent trials. The results provide strong evidence that a set of brain regions, including dMFC, is indeed involved in the processing of conflict for oculomotor responses irrespective of the time on task effect (Figure 4).

Our findings have two prominent indications. First, Grinband et al. (2011b) claim regarding dMFC activity being sensitive to time on task and not response conflict should be limited to ACC. The peak activation from an incongruent minus congruent contrast reported in their study was consistent with the mean location of conflict activation in meta-analysis study of Stroop task (Nee et al., 2007). However, Nee et al. (2007) refer to this location as ACC. Pre-SMA is a part of dMFC, yet we found its activity to be independent of time on task. With growing number of researches mapping the brain cognitive functions, the precision in neuroanatomy should be enhanced. Second, spatial cueing task with oculomotor responses provides a good model for studying motor conflict resolution due to the fact that conflict-related brain activations detected with fMRI are free of the “inconvenient” RT-BOLD correlations. Further studies using spatial cueing task with manual responses are recommended to address the significance of the response modality in those activations.

## Conclusion

The comparison of the incongruent and congruent trials obtained with the spatial cueing paradigm with oculomotor responses provided a suitable model for investigating the saccadic response conflict. The results revealed a set of brain regions involved in conflict resolution. Activities in bilateral IPS/precuneus, anterior IPS, FEF and AIC, as well as right DLPFC were associated with the requirement of additional processes in order to perform a correct response to incongruent stimuli. These processes are presumably the inhibition of the already programmed saccade and the vector inversion. Increased activity in pre-SMA was associated with resolving the response conflict by switching to a controlled alternative action.

Two approaches accounting for the time on task effect were applied to these conflict-related brain activations. Conducting the RT-regression analysis, following Carp et al. (2010), revealed a tendency toward the effect of response congruency being retained after controlling for RT. Applying the event-triggered averaging, following Grinband et al. (2011b), provided a strong evidence that conflict-induced brain activations are not sensitive to the time on task, but reflect purely the congruency effect. Our study highlights the importance of implementing saccadic task in the fMRI experiments and provides new insight into the neural mechanism of the performance monitoring and cognitive control.

## Conflict of Interest Statement

The authors declare that the research was conducted in the absence of any commercial or financial relationships that could be construed as a potential conflict of interest.
